# Water Splitting: From Electrode to Green Energy System

**DOI:** 10.1007/s40820-020-00469-3

**Published:** 2020-06-17

**Authors:** Xiao Li, Lili Zhao, Jiayuan Yu, Xiaoyan Liu, Xiaoli Zhang, Hong Liu, Weijia Zhou

**Affiliations:** 1 Collaborative Innovation Center of Technology and Equipment for Biological Diagnosis and Therapy in Universities of Shandong, Institute for Advanced Interdisciplinary Research (iAIR), University of Jinan, Jinan, 250022 People’s Republic of China; 2 State Key Laboratory of Crystal Materials, Shandong University, Jinan, 250100 People’s Republic of China; 3 School of Materials Science and Engineering, Zhengzhou University, Zhengzhou, 450001 People’s Republic of China

**Keywords:** Water splitting, Electrode, Green energy system, Renewable energy, Hydrogen production

## Abstract

Hydrogen (H_2_) production is a latent feasibility of renewable clean energy. The industrial H_2_ production is obtained from reforming of natural gas, which consumes a large amount of nonrenewable energy and simultaneously produces greenhouse gas carbon dioxide. Electrochemical water splitting is a promising approach for the H_2_ production, which is sustainable and pollution-free. Therefore, developing efficient and economic technologies for electrochemical water splitting has been an important goal for researchers around the world. The utilization of green energy systems to reduce overall energy consumption is more important for H_2_ production. Harvesting and converting energy from the environment by different green energy systems for water splitting can efficiently decrease the external power consumption. A variety of green energy systems for efficient producing H_2_, such as two-electrode electrolysis of water, water splitting driven by photoelectrode devices, solar cells, thermoelectric devices, triboelectric nanogenerator, pyroelectric device or electrochemical water–gas shift device, have been developed recently. In this review, some notable progress made in the different green energy cells for water splitting is discussed in detail. We hoped this review can guide people to pay more attention to the development of green energy system to generate pollution-free H_2_ energy, which will realize the whole process of H_2_ production with low cost, pollution-free and energy sustainability conversion.

## Highlights

- Bifunctional electrode and electrolytic cell configuration for electrochemical water splitting are reviewed.
- The different green energy systems powered water splitting are summarized and discussed.
- An outlook of future research prospects for the development of green energy system powered water splitting in practical application process is proposed.

## Graphical abstract

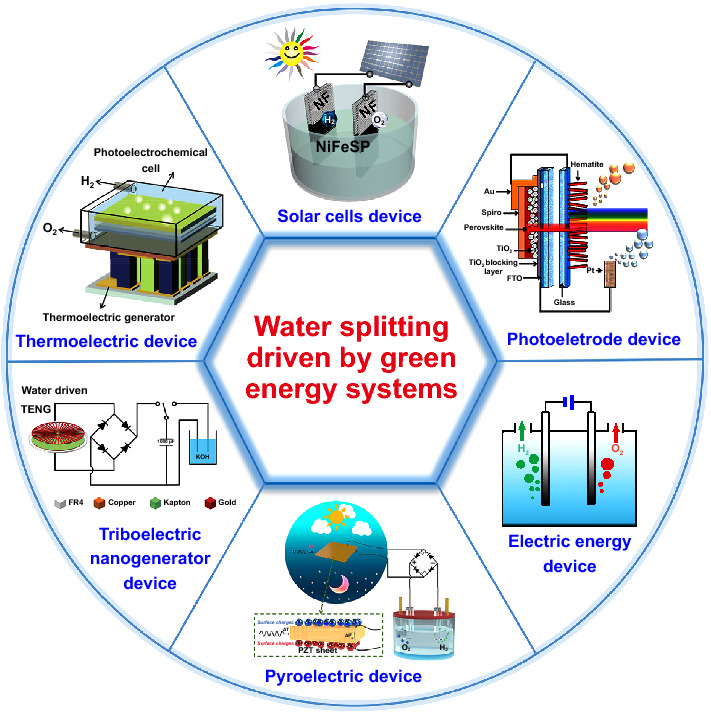

## Introduction

With the gradual intensity of global energy crisis, hydrogen (H_2_) is one of the most sustainable and clean energies for replacing fossil fuel energy [1, 2]. Reforming natural gas to produce H_2_ not only consumes a large amount of natural resources but also produces undesired carbon dioxide, which causes greenhouse effect [3–5]. Splitting water into H_2_ and oxygen (O_2_) was from more than 200 years ago. It is very important to develop an environmental-friendly and low-cost technology for large-scale production of H_2_ [6]. As a mature energy conversion technology, electrolysis of water provides a simple, efficient and promising method for the hydrogen evolution reaction (HER) [7–10]. However, an external power supply to deliver oxidation or reduction reactions of H_2_O is necessary for electrolysis, leading to economically inefficient application of energy. Alternatively, harvesting, storing and converting energy from the environment (such as wind, thermal, sunlight, tidal and self-powdered energy) [11, 12] can be directly utilized for electrolysis with using a lower or no external power supply.

Sunlight is an inexhaustible renewable energy source that can meet humanity’s needs. Effective utilization of solar energy can reduce the overall energy consumption of water splitting [13, 14]. For example, constructing a photoelectrode to absorb sunlight can provide a photovoltage to effectively reduce the external energy supply for electrolysis of water [15, 16]. In addition, solar cell is also an effective technology of solar energy conversion, which can directly absorb sunlight to transform output voltage instead of external electric energy, thus effectively realizing the minimum of external energy consumption. The utilization of heat energy from sunlight in nature for thermoelectric (TE) device can generate power to provide the voltage of water splitting [17]. There are also vast amount of wind and tidal energy in nature, which can be captured by triboelectric nanogenerator (TENG) to generate electricity, which can also effectively reduce the input of external energy. Therefore, it is of great significance to establish a suitable externally driven system of water splitting to reduce external consumption and improve H_2_ production capacity.

**Fig. 1:**
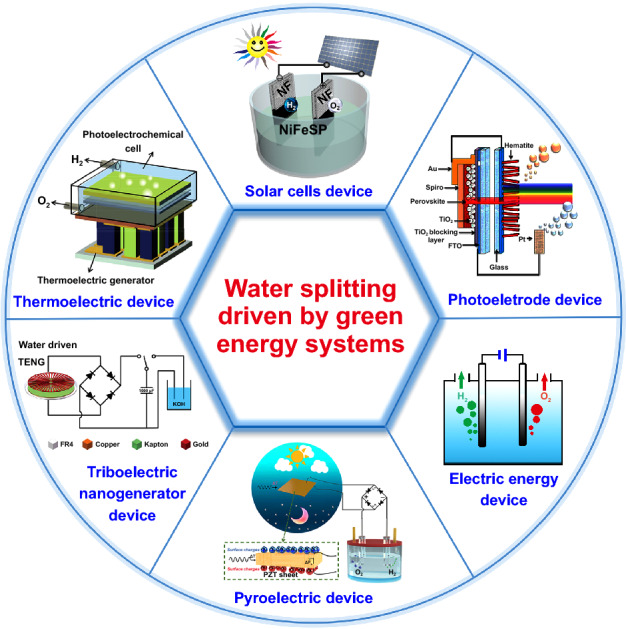
Water splitting driven by different green energy systems

For the past few years, many researchers have developed a variety of green energy system for efficient producing H_2_, such as two-electrode electrolysis of water, water splitting driven by a photoelectrode device, solar cells, TE device, TENG and other devices including pyroelectric and water–gas shift (WGS) reaction and so on (Fig. 1). These green energy systems can efficient drive water splitting for H_2_ production. Some notable matters and challenge in the different green energy system for water splitting are discussed in detail in this review.

## Two-Electrode Electrolysis of Water

Electrochemical water splitting is a prospective method to produce environmentally friendly hydrogen fuel [18]. Electrochemical water splitting requires a voltage of 1.23 V in theory; however, over 1.8 V is needed in practice to overcome the activation barrier of the reaction [19]. The large overpotential is from the slow four-electron transfer kinetics of the anodic oxidation reaction and the easy two-electron transfer kinetics of the cathode reduction reaction [20, 21]. In addition, it is difficult to establish a water splitting system of different cathode and anode because different catalysts are active and stable in different pH ranges. Moreover, the use of different catalysts in the same system often needs different equipment and methods, which increases the complicacy and cost of the system. And also, the wettability of the electrocatalyst with electrolyte and the rapid desorption of bubbles generated on the electrodes are very important in the process of water splitting [22–24]. If the generated gas bubbles are difficult to break away from the surface of electrode, the active site of electrocatalyst will be covered as well as the electrolyte will be difficult to diffuse to access the interface of catalyst/electrolyte [25]. Therefore, the hydrophilicity and aerophobicity of the electrode is very significant to promote the efficiency and stability of the water splitting process [26, 27]. Hence, the development of a high active, stable and low-cost bifunctional electrocatalyst for water splitting is imperative [28].

### Electrocatalysts for Overall Water Splitting

For overall water splitting, an ideal bifunctional electrocatalyst should be a low-cost, highly active and economical preparation method, which can provide long-term stability for both HER and oxygen evolution reaction (OER) in the electrolyte [29]. The employment of suitable catalyst will be critical to develop electrolysis of water. Hence, it is an urgent desire for researchers to develop many different kinds of bifunctional electrocatalysts with different performance to promote the development of H_2_ fuels [30].

**Fig. 2:**
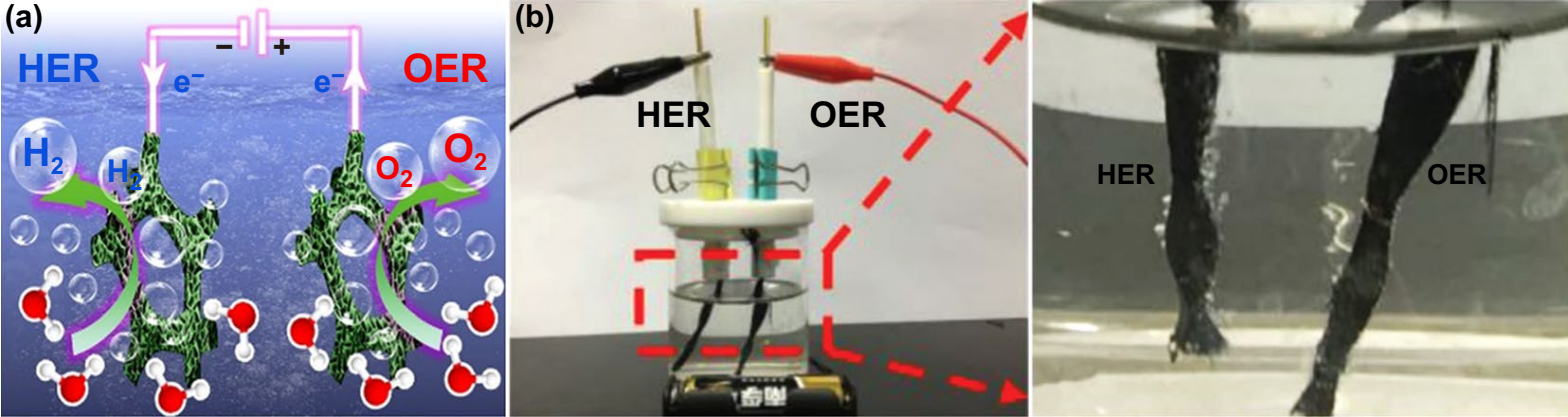
**a** Schematic diagram of two-electrode configuration for overall water splitting with NF/T(Ni_3_S_2_/MnS–O) as anode and cathode. Reproduced with permission from Ref. [33]. Copyright 2019 Elsevier Inc., **b** photographs showing the NiCo-nitrides/NiCo_2_O_4_/GF||NiCo-nitrides/NiCo_2_O_4_/GF couple electrolyzer. Reproduced with permission from Ref. [53]. Copyright 2019 John Wiley and Sons

Transition metal oxide [31, 32], transition metal sulfides [33–39] and selenides [40–43], transition metal phosphides [44–49], transition metal nitrides [50–52] become potential candidates as non-noble metal electrocatalysts for electrolysis of water. The Ni_3_S_2_/MnS–O nanosheets on Ni foam (NF/T(Ni_3_S_2_/MnS–O)) were employed as anode and cathode for overall water splitting (Fig. 2a), which was required a voltage of 1.54 V at a current density of 10 mA cm^−2^ [33]. Dai and Liu et al. prepared NiCo-nitrides/NiCo_2_O_4_/GF as both anode and cathode in two-electrode system; the whole voltage for electrochemical water splitting was 1.68 V to achieve 20 mA cm^−2^ in 1.0 M KOH (Fig. 2b) [53]. He and Sun et al. synthesized a bifunctional catalyst for electrolysis of water based on three-dimensional (3D) self-supported Fe-doped Ni_2_P nanosheets on NF. An two-electrode electrolyzer composed of the (Ni_0.33_Fe_0.67_)_2_P||(Ni_0.33_Fe_0.67_)_2_P electrodes required a low cell voltage of 1.49 V to achieve 10 mA cm^−2^ in 1.0 M KOH [45]. Wang et al. reported that the nanostructured porous Ni_3_FeN nanosheet was obtained by annealing process the Ni_3_Fe LDHs precursor in NH_3_ atmosphere. The porous Ni_3_FeN used as both anode and cathode in two-electrode system for overall water splitting in 1.0 M KOH required a voltage of 1.495 V at 10 mA cm^−2^, which could be driven by a battery with rated voltage of 1.5 V [50]. Metal-free electrocatalysts also show high activity, good stability and low cost to replace metal-based electrocatalysts for long-term water splitting [54, 55]. Yu, Chen, Dai et al. reported a novel metal-free bifunctional electrocatalyst with the ultrathin exfoliated black phosphorus (EBP) nanosheets on N-doped graphene (EBP@NG). EBP@NG possessed excellent performance of HER and OER in 1.0 M KOH. The voltage of an optimized two-electrode cell with EBP@NG used as anode and cathode was 1.54 V to achieve 10 mA cm^−2^ [54]. The voltage of most reported bifunctional non-noble metal electrocatalysts is lower than that of benchmarking IrO_2_||Pt electrodes (1.57 V at 10 mA cm^−2^) and standard coupled Ni and stainless steel (1.73 V at 10 mA cm^−2^) in the industrial application [56]. A detailed comparison of the HER and OER activities of recently reported electrocatalysts for overall water splitting are listed in Table 1.

**Table 1:** Summary of the HER and OER activities of recently reported electrocatalysts for overall water splitting

Catalysis	Electrolytes	*ƞ* for HER at *j* (mV@mA cm^−2^)	*ƞ* for OER at *j* (mV@mA cm^−2^)	Tafel slope for HER (mV dec^−1^)	Tafel slope for OER (mV dec^−1^)	Overall voltage at *j* (V@mA cm^−2^)	References
Co_3_O_4_@C@NF	1.0 M KOH	42@10	96@10	56	89	1.40@10	Ha et al. [31]
NF/H–CoMoO_4_	1.0 M KOH	295@10	–	91	–	1.56@10	Chi et al. [32]
NF/T(Ni_3_S_2_/MnS–O)	1.0 M KOH	116@10	228@10	41	46	1.54@10	Zhang et al. [33]
N–CoS_2_/NF	1.0 M KOH	28@10	200@20	42.6	55	1.50@10	Yao et al. [34]
MoS_2_/NiS	1.0 M KOH	244@10	370@11	97	108	1.64@10	Qin et al. [35]
MoS_2_–Ni_3_S_2_ HNRs/NF	1.0 M KOH	98@10	314@10	61	57	1.50@10	Yang et al. [36]
Ni_3_S_2_/NF	1.0 M KOH	189@10	296@10	89.3	65.1	1.55@10	Li et al. [37]
MoS_2_/NiS_2_	1.0 M KOH	62@10	278@10	50.1	91.7	1.59@10	Lin et al. [38]
Ni_3_Se_4_@NiFe LDH/CFC	1.0 M KOH	85@10	223@10	98.6	55.5	1.54@10	Zhang et al. [40]
CoSe@NiFe LDH/NF	1.0 M KOH	98@10	201@10	89	39	1.53@10	Sun et al. [41]
Co–Ni–Se/C/NF	1.0 M KOH	90@10	275@30	81	63	1.6@10	Ming et al. [42]
MoSe_2_/MXene	1.0 M KOH	95@10	340@10	91	90	1.64@10	Li et al. [43]
CoP_2_/RGO	1.0 M KOH	88@10	300@10	50	96	1.56@10	Wang et al. [44]
(Ni_0.33_Fe_0.67_)_2_P	1.0 M KOH	214@50	230@50	–	55.9	1.49@10	Li et al. [45]
NF@Fe_2_–Ni_2_P/C	1.0 M KOH	39@10	205@10	30	52	1.57@100	Sun et al. [46]
NiCoP@NC NA/NF	1.0 M KOH	37@10	305@50	53.9	70.5	1.56@20	Cao et al [47]
CoFeP TPAs/Ni	1.0 M KOH	43@10	198@10	65.3	42	1.47@10	Zhang et al. [48]
Mo–NiCoP	1.0 M KOH	76@10	269@10	60	76.7	1.61@10	Lin et al. [49]
Ni_3_FeN	1.0 M KOH	45@10	223@10	75	40	1.495@10	Wang et al. [50]
Ni_3_N–NiMoN	1.0 M KOH	31@10	277@10	64	118	1.54@10	Wu et al. [51]
CoAl–Fe_2_N/Fe_3_N	1.0 M KOH	145@10	307@10	54	69	1.67@10	Hu et al. [52]
NiCo-nitrides/NiCo_2_O_4_	1.0 M KOH	71@10	183@10	41	54	1.68@20	Liu et al. [53]
EBP@NG	1.0 M KOH	125@10	265@10	76	89	1.54@10	Yuan et al. [54]

### Electrolytic Cell

Conventional water electrolysis usually utilizes transition metal catalysts and diaphragms in alkaline electrolytes (alkaline water electrolysis, AWE) or noble metal catalysts and a proton exchange membrane in acidic media (PEM water electrolysis) [57, 58].

#### Alkaline Water Electrolysis

Since Troostwijk and Diemann first found the phenomenon of electrolysis of water in 1789, alkaline water electrolysis has been an established technique for H_2_ production. Therefore, alkaline electrocatalysis is the most widely used electrolysis technology on a business level in the world [59–64]. In the AWE, the electrolyte is made up of a caustic potassium solution with a concentration of 20–30% KOH [65–67]. The configurations of alkaline electrolyzer contain the conventional alkaline electrolyzer, the “zero-gap” alkaline electrolyzer and the membraneless or decoupled alkaline electrolyzer.

**Fig. 3:**
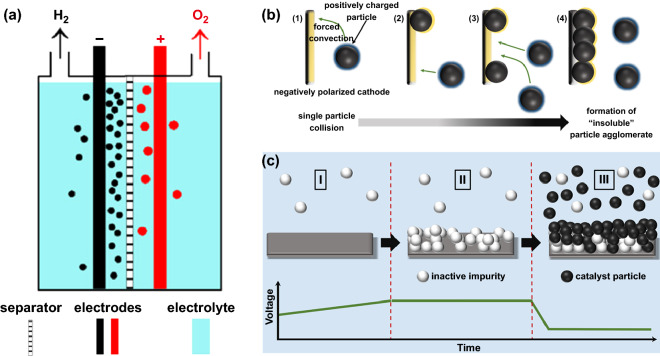
**a** Schematic diagram of conventional alkaline electrolyzer. Reproduced with permission from Ref. [68]. Copyright 2012 Elsevier Inc., **b** schematic diagram of the formation of the catalyst film, **c** schematic diagram of cathode deactivation caused by the deposition of trace metal impurities and the change of the overall voltage in the electrolytic cell. Reproduced with permission from Ref. [69]. Copyright 2018 Elsevier Inc

In conventional alkaline electrolyzer, the anode and cathode immersed in the electrolyte are located on either side of the flat current collector to facilitate a serial connection between cells [68]. H_2_ and O_2_ bubbles are formed in two electrolyte chambers; meanwhile, a membrane avoids the mixture of them (Fig. 3a). This method is easy to scale up to the massive volume production of H_2_. However, the resulting bubbles decrease the effective area of the electrodes and improve the resistance of the electrolytes, leading to low current densities. Another issue of AWE of Ni-based electrodes is the sustaining attenuation of the activity for HER and OER. The deposition of metal cations mainly from electrolyte impurities can generate a surface coating with low catalytic activity, leading to reducing the activity for HER. Therefore, Schuhmann and Ventosa et al. creatively brought forward a method based on in situ self-assembly of catalyst particles in the electrolytic process to obtain exceptionally stable catalytic films with the capability of self-healing (Fig. 3b, c). They showed that the passivation of cathode by zinc impurities from the electrolyte could be surmounted by immobilizing catalyst with self-assembly and self-healing films. In the electrolytic process, zinc impurities deposited on the cathode electrode in the form of a dendritic film increased the HER overpotential, but the continued self-assembling and self-healing of the catalyst films following obscured the zinc dendrites that restored the favorable overpotential of the HER [69].

**Fig. 4:**
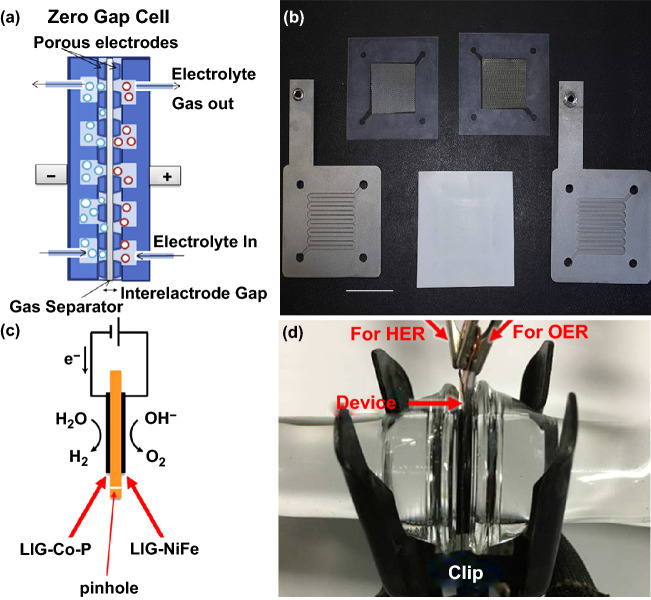
**a** Schematic diagram for reducing the gap between electrodes by using a zero-gap cell, **b** components for the zero-gap cell, including the machined flow field plates, silicone gaskets, mesh electrodes and Zirfon gas separator. Reproduced with permission from Ref. [70]. Copyright 2017 Elsevier Inc., **c** schematic diagram and **d** a photograph of an integrated LIG electrolyzer. Reproduced with permission from Ref. [72]. Copyright 2017 American Chemical Society

In the “zero-gap” configuration, a thin cellulose felt occupied the intra-electrode space can absorb the electrolyte, which is confined and clamped between two hydrophilic separators that are tightly pressed on the anode and cathode. The anode and cathode ought to be polyporous to permeate the liquid electrolyte. Therefore, the bubbles from the inner space of the electrode can be efficiently excluded [68]. For instance, Dunnill et al. reported that employing a zero-gap cell configuration could reduce 30% in ohmic resistance in comparison with the traditional a 2-mm gap in alkaline electrolyte (Fig. 4a, b). At all current densities, especially over 500 mA cm^−2^, the performance of zero-gap configuration cell was better than the standard cell. In addition, the foam electrodes with high surface area allowed for a low ohmic resistance compared to the coarse mesh electrodes. Therefore, the zero-gap configuration will permit low cost and high-efficiency alkaline electrolysis [70]. The anode and cathode also can be fabricated on the separators to further decrease the distance of the gap [71]. For instance, Tour et al. used the laser-induced graphene (LIG) to form HER and OER catalysts on each side of a polyimide (PI) film to assemble high-efficiency electrodes for electrolysis of water. In this alkaline electrolyzer, LIG was patterned on each side of a PI film and subsequently assembled LIG-Co–P and LIG-NiFe on opposite sides by electrochemical deposition (Fig. 4c, d). The hydroxide ions could migrate through a small pinhole at the end of the film that may be covered by ion exchange membranes for large-scale applications. As expected, the device of LIG-Co–P and LIG-NiFe for water splitting required 1.66 V to achieve a current density of 10 mA cm^−2^ in 1.0 M KOH [72].

**Fig. 5:**
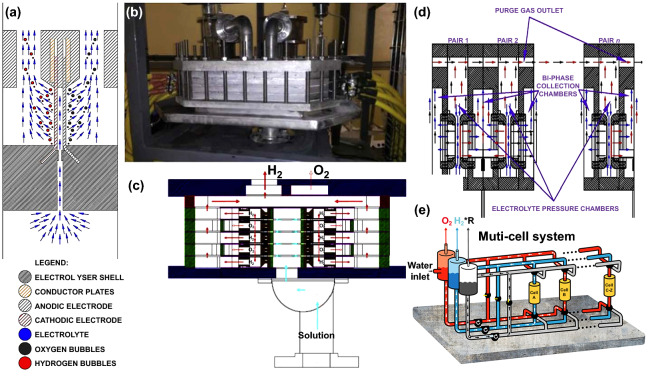
**a** Schematic diagram of filtration mesh electrode encapsulated in a single injection assembly, **b** a photograph of the horizontal filter press DEFT electrolyzer stack, and **c** a diagram of a cross section of the electrolyzer stack assembly. Reproduced with permission from Ref. [75]. Copyright 2017 Elsevier Inc., **d** cross section of the DEFT electrolyzer stack in a MCFP configuration. Reproduced with permission from Ref. [76]. Copyright 2018 Elsevier Inc., **e** schematic diagram of the multicell system by the E-TAC process. Reproduced with permission from Ref. [83]. Copyright 2019 Springer Nature

In alkaline water electrolysis, the conductivity of liquid electrolyte is much higher than that of the ion exchange membrane leading to significant ohmic losses [73, 74]. Therefore, Gillespie and Kriek constructed a membraneless DEFT alkaline electrolyzer for the gainful production of H_2_. The electrolyzer could overcome the limitation of current density threshold in the existing technology and was an ideal choice for H_2_ generation (Fig. 5a). The scale-up of the technology represented a difference from the design of original tested stack, which encapsulated many slender electrodes in a pressure filter assembly (Fig. 5b, c). The operation parameters of the pilot plant were limited to low flow rate, and the electrode gap was 2.5 mm. The performance of the pilot plant is consistent with the previous acquired results. The geometric area of mesh electrodes used for the performance test of plant was 344.32 cm^2^. Under the conditions of 0.04 m s^−1^, 30% KOH, 2 V direct current (VDC) and 80 °C, the best performance of the NiO anode and Ni cathode combination reached to 508 mA cm^−2^. Unfortunately, due to the nature of the gas–liquid separation system, the gas mass was insufficient in comparison with previous results [75].

To further promote the purities of the produced gas in the DEFT electrolyzer, Gillespie and Kriek developed an extensible and simple mono-circular filter press (MCFP) reactor for the DEFT alkaline electrolysis (Fig. 5d). Under the condition of the flow rate (0.075 m s^−1^) and electrode gap (2.5 mm), the utilized gas/liquid separation methodology improves the gas purities of H_2_ to 99.81 vol% and O_2_ to 99.50 vol%. Each round mesh electrode pair of 30 mm has independent pressurized chamber and indirect injection of the electrolyte. By incorporating a gas purge, the high gas purity could be kept for a long running time. Using a Ni/Ni catalyst, the current density was 1.14 A cm^−2^ (2.5 VDC) at a flow rate of 0.075 m s^−1^, 60 °C and 2.5-mm electrode gap. Under the same condition except the utilization of a double-layer mesh electrode, a current density reached to 1.91 A cm^−2^ at 2.5 VDC was realized, confirming that the multilayer microporous electrodes for the DEFT principle were available [76].

Utilizing decoupled the two half-reactions of water splitting by redox mediators can completely avoid the mixture of produced H_2_ and O_2_, which is promising for large-scale practical application [77–82]. Grader et al. proposed a two-step electrochemical-thermally activated chemical (E-TAC) cycle for overall water splitting. H_2_ was produced by a HER at the cathode. The traditional OER was replaced by two steps. In the first step, the Ni(OH)_2_ anode was oxidized to NiOOH by four one-electron oxidation reactions. In the second step, the oxidized NiOOH could be spontaneously reduced to Ni(OH)_2_ in an exergonic chemical reaction to simultaneously achieve O_2_ production and anode regeneration. As shown in Fig. 5e, they also assumed a multicell system with fixed anodes and cathodes in each cell for practical application to produce pure H_2_ and O_2_ gas. A low-temperature electrolyte flew through cell A, driving the produced H_2_ to the H_2_ separator. Meanwhile, a high-temperature electrolyte flew through cell B to regenerate the anode, driving the generated O_2_ to the O_2_ separator. In this multicell system, only hot and cold electrolyte moved in the operation process [83].

#### PEM Water Electrolysis

In 1960s, General Electric firstly proposed a concept of solid polymer electrolyte (SPE) concept for water electrolyzer, which predicted to conquer the disadvantages of alkaline electrolyzers. Grubb idealized the above concept by using solid sulfonated polystyrene membranes as the electrolytes, also known as PEM water electrolysis, rarely called SPE water electrolysis [84, 85]. The polymer electrolyte membrane could provide higher proton conductivity, lower gas exchange, the compact design of system and operate under high pressure [86–90]. The advantages of solid polymer electrolyte were lower membrane thickness (~ 20–300 μm thick).

**Fig. 6:**
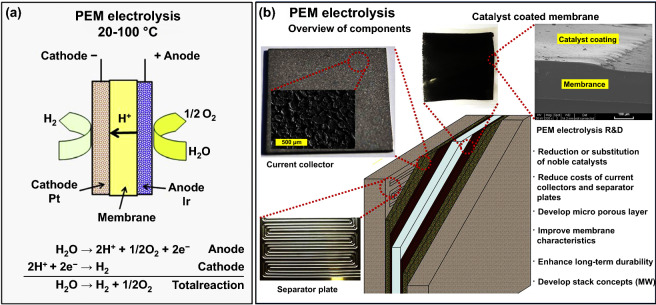
**a** Schematic diagram of the working principle and **b** composition of a PEM water electrolysis. Reproduced with permission from Ref. [65]. Copyright 2013 Elsevier Inc

First of all, the catalyst layer was coated on a glossy polytetrafluoroethylene (PTFE) sheet; then, the coating consisted of catalyst and Nafion ionomer on the surface of a Nafion 117 membrane was flat when the PTFE sheet was removed after being pressed against the membrane. The edge between the catalyst coating and membrane was obvious on the surface of membrane. The thickness of the coating could change by adjusting the amount of catalyst ink [91]. In commercial PEM water electrolyzer, a layer consisted of Pt/C and Nafion ionomer, similarly a layer of made up of IrO_2_ or RuO_2_ catalyst and Nafion ionomer, was coated on the opposite side of a Nafion 117 membrane, respectively (Fig. 6a).

The anode side of the PEM water electrolyzer was filled with water. Water successively passed through the separator plates and current collectors. When water got to the surface of catalyst layer, the molecules of water were broken up into protons, electrons and diatomic oxygen. Subsequently, the generated protons left the anode through the ionomer and the membrane, passing through the side of the cathode, where they coupled with electrons to form H_2_ after they arrived the catalytic layer. Then the H_2_ must flow through the cathode collector and the barrier, away from the cell. At the same time, the electrons left the cathode catalytic layer via the current collector, the separator plates, then departured to the side of the cathode. O_2_ must flow back via the catalyst layer and current collector to the separator plates then go out of the cell (Fig. 6b) [65].

The PEM electrolyzers could work at high current densities of over 2 A cm^−2^, reducing the operating cost and the potential total cost of water electrolysis. The ohmic losses confined the maximum value of current densities. The thin membrane capable of providing good proton conductivity and high current densities could be achieved. The low gas crossover rate of the polymer electrolyte membrane allowed for the PEM electrolyzer to operate at a wide range of power input.

The phenomenon of cross-permeation enlarged along with high operational pressure in PEM electrolysis [92]. High pressures (over 100 bar) required thicker membranes to reduce the mixture of H_2_ and O_2_, which kept the marginal concentrations below the safety threshold (4 vol% H_2_ in O_2_) [92]. The corrosive acidic regime in the PEM electrolysis required distinct materials, which needed the resistance to severe corrosive low pH corrosion (pH ~ 2) and kept at high overvoltage (~ 2 V).

#### Seawater Electrolysis

Water electrolysis systems usually consist of two half-reactions: HER at the cathode and OER at the anode. Compared to the limited pure water, seawater is the most abundant aqueous electrolyte on earth for the utilization in the process of water electrolysis. Seawater electrolysis was investigated by Bennett [93], which was composed of HER at the cathode and chlorine evolution reaction (ClER) at the anode [66]. ClER is a two-electron process, and chlorine or hypochlorite is the value-added product [94]. Four years later, Trasatti used different anodes for seawater electrolysis to investigate the selectivity for anodic process [95]. In 2016, Dionigi et al. proposed the chemical limitations of seawater electrodes and presented the design criterion for selective seawater splitting catalysts [96].

**Fig. 7:**
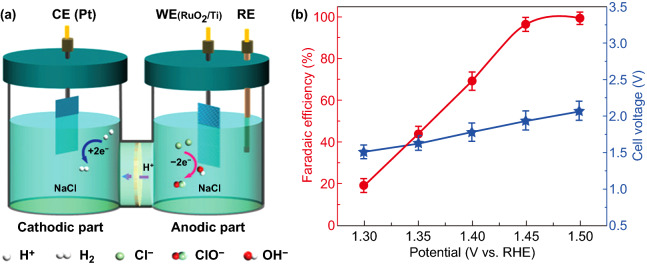
**a** Schematic of the electrocatalytic cell for cathodic HER and anodic hypochlorite production, **b** dependence of the FE of hypochlorite with the applied potentials on the cathode and the cell voltage. Reproduced with permission from Ref. [97]. Copyright 2019 Royal Society of Chemistry

In the seawater electrolysis system, the membrane (such as Zirfon) should be physically robust and insusceptible to blockages, because largely block either anions or cations (such as H^+^, Na^+^, OH^−^ and Cl^−^ and so on) are able to migrate through the membrane [66]. A commercial ruthenium oxide-coated titanium electrode (RuO_2_/Ti) and Pt electrode were served as the working electrode and the counter electrode, respectively, to achieve ClER at the anode and HER at the cathode (Fig. 7a). The FE of hypochlorite increased linearly with the applied potential on the anode, which could achieve 99% at the applied potential of 1.5 V vs. RHE on the RuO_2_/Ti electrode (Fig. 7b) [97].

## Water Splitting Driven by a Photoelectrode Device

Electrolyzed water can effectively generate H_2_ through a two-electrode system. However, it takes a large amount of electrical energy to conquer the thermodynamic barrier in the electrolysis of water. In the photoelectrochemical (PEC) electrolysis cell [98, 99], the photoanode absorbs the solar energy to generate the photovoltage to effectively drive water splitting, which can effectively decrease the external energy consumption [100–102]. To minimized utilization of the external energy consumption and realize unassisted overall light-induced water splitting, a possible way is using a tandem structure to generate a total photovoltage through complementary light absorption between different semiconductor electrodes [103–113].

**Fig. 8:**
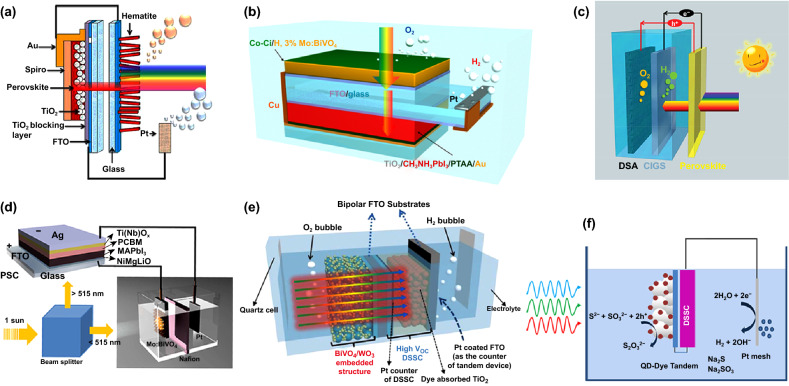
**a** Schematic of the dual-junction PSC/hematite photoanode tandem cell. Reproduced with permission from Ref. [114]. Copyright 2015 American Chemical Society, **b** configuration consisted of Co–Ci/H, 3% Mo:BiVO_4_ and TiO_2_/CH_3_NH_3_PbI_3_ tandem cell. Reproduced with permission from Ref. [115]. Copyright 2015 American Chemical Society, **c** schematic of the perovskite and CIGS tandem water splitting cell. Reproduced with permission from Ref. [116]. Copyright 2015 John Wiley and Sons, **d** the configuration of the PEC-PSC tandem device. Reproduced with permission from Ref. [117]. Copyright 2016 American Association for the Advancement of Science, **e** schematic of the WO_3_/BiVO_4_ photoanode tandem configuration. Reproduced with permission from Ref. [120]. Copyright 2015 Elsevier Inc., **f** scheme of the PEC-DSSC tandem cell. Reproduced with permission from Ref. [121]. Copyright 2013 American Chemical Society

Mathews et al. constructed that a Fe_2_O_3_ photoanode in tandem with an organic–inorganic CH_3_NH_3_PbI_3_ perovskite solar cell (PSC) (Fig. 8a) could achieve overall unassisted water splitting at air mass 1.5 global (AM 1.5G) irradiation with a solar-to-hydrogen (STH) conversion efficiency of 2.4%. The total potential produced by this tandem system reached to 1.87 V, which was surpassed the required thermodynamic and kinetic potential of 1.6 V, delivering water splitting with no external energy consumption [114]. Jun and Lee et al. reported that cobalt carbonate-catalyzed, H and 3 at% Mo dual-doped BiVO_4_ (Co–Ci/H, 3% Mo:BiVO_4_) device in series with CH_3_NH_3_PbI_3_ single-junction PSC could realize wireless solar water splitting under AM 1.5G without external energy supply (Fig. 8b). The STH efficiency of the device exhibited STH efficiency was 3.0%, which could be even higher along with the improvement of the photoanode performance [115]. Luo et al. reported that a semilucent CH_3_NH_3_PbBr_3_ PSC as the top absorber pairing with a CuIn_*x*_Ga_1−*x*_Se_2_ (CIGS) multilayer photocathode as the bottom absorber could panchromatic harvest of the solar spectrum for effective overall water splitting (Fig. 8c). For this PV-PEC system employing a single-junction PSC as the bias source at AM 1.5G irradiation, a STH efficiency was reached to over 6%. Moreover, the efficiency could attain over 20% by further optimizing the performance of the perovskite top absorber [116]. Qiu et al. constructed a single PSC in tandem with nanoporous Mo-doped BiVO_4_ (Mo:BiVO_4_) photoanode PEC cell device by using a beam splitter to divide a standard sunlight beam into two light beams (Fig. 8d). The PSC-PEC serial system achieved unassisted water splitting with a STH efficiency of 6.2% and long-term stability over 10 h (only 5.8% decay) [117].

Owing to the energy supply is efficient and the materials (e.g., TiO_2_) are cheap, abundant and environmentally friendly, dye-sensitized solar cell (DSSC) in tandem with photoelectrodes are prospective system for unassisted water splitting [118]. Herein, Sivula et al. constructed a device based on an oxide (WO_3_ or Fe_2_O_3_) photoanode in series with a DSSC for unassisted water splitting. In this device, the light was incident on the photoanode before the underlying DSSC. The WO_3_/DSSC serial system reached a STH conversion efficiency of 3.10%, while that of 1.17% in the Fe_2_O_3_/DSSC tandem device. For the two tandem cells, the optical transmittances and spectral responses matched with the bandgaps of oxide, determining the photocurrent and performance of devices. The performance of Fe_2_O_3_/DSSC PEC tandem cells was retained 80% after more than 8 h, which attributed to the degradation of DSSC. Therefore, the layout relied on chosen redox mediators and catalysts for the DSSC and photoanodes, respectively [119]. Wang and Park et al. demonstrated a 5.7% STH without any external bias unassisted monolithic tandem system, which was combined the high transparency of BiVO_4_-sensitized mesoporous WO_3_ films/Pt with a single DSSC (Fig. 8e). On one hand, the BiVO_4_ coating on the porous WO_3_ films maintained the high transparency, allowing enough photons to enter the dye-sensitized photoanode. On the other hand, the porphyrin-dye-sensitized photoanode with a cobalt electrolyte produced enough potential to achieve wireless solar water splitting in the tandem system [120]. Mora-Sero and Gimenez et al. established a tandem device combined a CdS quantum dots modified TiO_2_ photoanode connected with a DSSC for water splitting with no external bias (Fig. 8f). This device showed a STH conversion efficiency of around 0.78% and high stability. Designing hybrid photoanodes with different light absorbers was important for developing efficient water splitting devices [121].

## Water Splitting Driven by Solar Cells

As energy storage systems, solar cells, including Si solar cells, CIGS solar cells, PSCs, organic solar cells (OSCs) and DSSCs [104, 122–124], are able to transform surplus solar energy into storable and distributable energy carriers. The photovoltage of series connected solar cells can drive water electrolysis [125].

### By Conventional Solar Cells

For photovoltaic (PV)-driven water splitting, several connected crystalline conventional solar cells (such as Si and CIGS solar cells) are prospective because of the high STH efficiency and solar-driven durability for H_2_ production [60, 104, 126].

**Fig. 9:**
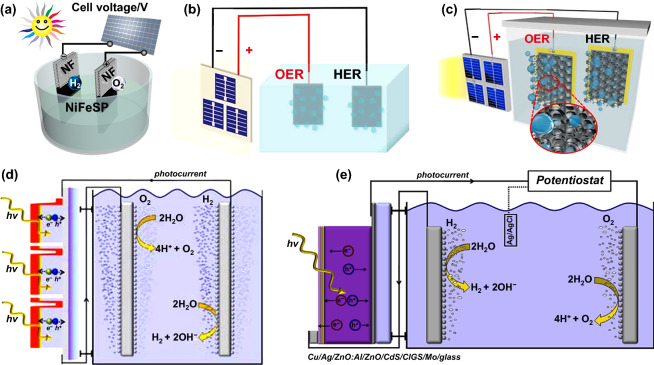
**a** Schematic diagram of the solar-driven system with a Si solar cell for overall water splitting. Reproduced with permission from Ref. [127]. Copyright 2017 American Chemical Society, **b** unassisted water splitting using NCS/NCP/NF as the electrodes and Si solar cells as the light absorber. Reproduced with permission from Ref. [122]. Copyright 2018 Royal Society of Chemistry, **c** unassisted water splitting by using a combination of an NiFe electrolyzer and Si solar cells. Reproduced with permission from Ref. [128]. Copyright 2017 Elsevier Inc., **d** schematic diagram of the CIGS absorber connected with the catalyst module by wires. Reproduced with permission from Ref. [132]. Copyright 2013 Royal Society of Chemistry, **e** schematic diagram of the CIGS electrode connected with a working electrode with deposited catalyst in the electrolyte by wires. Reproduced with permission from Ref. [133]. Copyright 2013 Elsevier Inc

Gan and Zhang et al. proposed a bimetallic compound NiFeSP on the commercial NF (NiFeSP/NF) in series with a Si solar cell to implement overall water splitting (Fig. 9a). The voltage of combination of the Si solar cell and the bifunctional NiFeSP/NF electrodes for water splitting in the tandem system was 1.58 V to reach a current density of 10 mA cm^−2^, corresponding to a STH conversion efficiency of ~ 9.2% [127]. Shen et al. also reported that three Si solar cells in series (entire area of 3 cm^2^) were combined with the double-layer Ni–Co–S/Ni–Co–P electrocatalyst on NF (NCS/NCP/NF) electrodes for unassisted water splitting (Fig. 9b). When using NCS/NCP/NF as a bifunctional catalyst for water splitting, the current density of 10 mA cm^−2^ can be obtained with only 1.49 V. Finally, the whole solar water splitting was realized with the efficiency of STH reached to 10.8% [122]. Oh, Ryu and Kim et al. combined four Si heterojunction solar cells in series with a bifunctional NiFe nanostructures electrocatalyst to realize water splitting (Fig. 9c). The overpotential of NiFe inverse opal electrolyzer for water splitting was ~ 160 mV, achieving a STH conversion efficiency of 9.54% more than 24 h with no bias condition [128].

Compared to Si solar cell, the outstanding advantage of CIGS is that the band gap energy can be modulated to effectively absorb the solar spectrum, so it is also widely used to achieve water splitting [129–131]. For purpose of overcoming the problem of low energy to drive overall water splitting, connected series into a monolithic device can be adopted to supply enough to drive the whole reaction. For example, Jacobsson et al. reported that three series-interconnected compound semiconductor CIGS PV electrolysis could efficiently realize solar water splitting at AM 1.5G irradiation (Fig. 9d). The current density was centered at 8.5 mA cm^−2^ and a STH conversion efficiency reached to 10.5% [132]. Jacobsson et al. demonstrated CIGS solar cell could be applied to water splitting into H_2_. They used a *p*–*n* junction for separating the charge and a catalyst deposited on the surface to significantly improve the performance in the configuration of a PEC cell (Fig. 9e). In this device, the efficient charge separation production from the catalysis improved the durability of CIGS in the light irradiation. Furthermore, photocurrents in this device could reach to over 20 mA cm^−2^. The full potential of CIGS as a highly efficient absorbent material for water cracking was demonstrated. They confirmed the full potential of CIGS as a highly effective absorbent material could be used for water splitting [133].

### By Perovskite Solar Cells

**Fig. 10:**
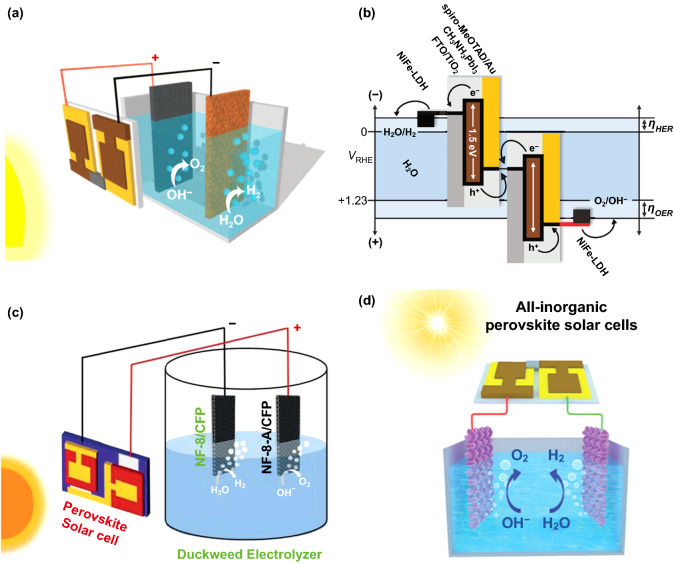
**a** Schematic diagram and **b** a generalized energy of the tandem PSC for water splitting. Reproduced with permission from Ref. [140]. Copyright 2014 American Association for the Advancement of Science, **c** schematic diagram of the solar energy-driven overall water splitting device with a PSC. Reproduced with permission from Ref. [141]. Copyright 2018 Royal Society of Chemistry, **d** configuration of the electrolyzer with the Ni_0.5_Co_0.5_P/CP bifunctional electrocatalyst and all-inorganic PSCs under irradiation. Reproduced with permission from Ref. [123]. Copyright 2018 Royal Society of Chemistry

In the above tandem system for water splitting in Sect. 4.1, owing to the low open-circuit voltages of Si solar cells, at least three to four connected cells in series must be utilized to achieve reasonable efficiency. In contrast, PSCs have achieved open-circuit voltages at 0.9 V and up to 1.5 V [134–139], which is sufficient for efficient water splitting by connecting just two in series [123]. Grätzel reported that the tandem PSC could be used to drive electrolytic splitting of water. The configuration was a water splitting system combing with a solution-processed tandem PSC and NiFe LDH used as anode and cathode electrodes in alkaline electrolyte (Fig. 10a, b). A photocurrent density of the tandem two-electrode system was around 10 mA cm^−2^, corresponding to a STH efficiency of 12.3% [140]. Bhattacharyya et al. developed NiFe-alloy nanoparticles supported by N, S-doped mesoporous carbon matrix from duckweed as efficient electrocatalysts (Fig. 10c). For overall water splitting, only 1.61 V was required to attain a current density of 10 mA cm^−2^ for over 200 h. Combining with PSCs, the electrolyzer for overall water splitting showed a STH efficiency of 9.7%, which is completely powered by solar energy [141]. Jin et al. reported that bifunctional bimetallic phosphide (Ni_0.5_Co_0.5_P/CP) in tandem with all-inorganic PSCs (based on a CsPb_0.9_Sn_0.1_IBr_2_ light absorber and a nanocarbon electrode Fig. 10d) realized efficient overall water splitting. The water splitting electrolyzer could achieve a current density of 10 mA cm^−2^ at only 1.61 V. Driven by stabilized all-inorganic PSCs, the electrolyzer delivered a STH conversion efficiency of 3.12% and good durability [123].

## Water Splitting Driven by Thermoelectric Device

Water splitting driven by solar cell is a common energy-driven water splitting strategy. However, the utilization efficiency of sunlight by the solar cell is relatively low because solar cells are chiefly effective in the range of ultraviolet and visible light. Conventional semiconductor solar energy conversion technology cannot efficiently utilize the infrared light, which occupies nearly half of the sunlight. The combination of TE device and infrared-active materials supplies a particular approach to transform infrared sunlight to the electricity, which improves the solar energy utilization efficiency [142–147]. Thus, the study of water splitting driven by TE device is extensively performed.

### By Surface-Modified Thermoelectric Device

**Fig. 11:**
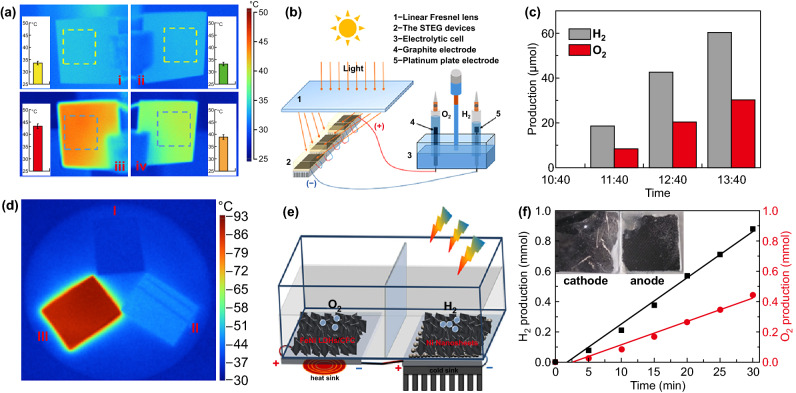
**a** Temperature distributions under 1 sun irradiation of: (i) hot side and (ii) cold side of bare generator; (iii) hot side and (iv) cold side of CNP generator. Inserts: temperature statistics of the area in corresponding dotted box, **b** schematic of the overall water splitting system driven by 6 series-wound STEG devices, **c** production of H_2_ and O_2_ by the water splitting system at different time. Reproduced with permission from Ref. [156]. Copyright 2018 Elsevier Inc., **d** the infrared thermal images of Al_2_O_3_ ceramic chip (I) and Al_2_O_3_ ceramic chip with Ni film (II) and Ni nanosheets/Ni film (III), **e** illustration of electrolyzer-TE hybrid device, **f** H_2_/O_2_ production versus time curve of electrolyzer-TE hybrid device with additional 0.7 V applied voltage. Reproduced with permission from Ref. [157]. Copyright 2019 Elsevier Inc

The infrared light usually delivers the energy in the form of heat through photothermal effect [148, 149]. Transforming the released heat into available energy (e.g., electricity) is a distinct method to utilize the infrared light. This conversion can be probable realized by TE device [150–152]. It is a pity that the surface of commercial TE device is unserviceable to absorb the infrared light. In order to improve the efficiency of TE devices, it is necessary to expand the absorption of infrared light by TE devices [151]. This requirement enlightened us to develop the probability of integrating photothermal materials on the TE device to promote the efficiency of photo-thermoelectric conversion [153]. Generally, materials with higher photothermal conversion efficiency include the Group VIII metal materials, graphene oxide (GO) [154], carbon nanomaterials, transition metal oxides (e.g., MoO_2_, WO_3_, Fe_2_O_3_) and chalcogenides (e.g., Cu_2_S) [155]. In 2014, the photothermal effect of GO had been demonstrated for the first time for TE devices [153]. The GO drop-coated on the surface of the TE device could transform the infrared light to the electricity, which was directly utilized to carry out photoelectrocatalytic process in the case of no applied voltage. As could be seen from the infrared thermal image, the conclusion that the surface coating of GO could significantly increase the response of TE devices can be drawn. Our group employed carbon nanoparticles (CNP) light absorbent layer on the top to increase the absorption efficiency of STEGs. A very easy candle flame preparation method was employed to synthesize the black CNP layer on the hot end of the commercial TE device (CNP generator). The synthesized CNP layer had a 3D porous structure which was conducive to capture light, and the power produced by this STEG device could drive an electrolyzer for splitting water to produce H_2_ (Fig. 11a, b). In this water splitting system driven by TE device, 6 sets of CNP coated thermoelectric generator devices were series connection to supply adequate voltage for electrolysis of water. After connecting the TE generator with the electrochemical cell, the cathode and anode immediately generated plenty of bubbles under the sunlight irradiation. The production of H_2_ and O_2_ was at an average rate of 20 and 10 μmol h^−1^, respectively, and the rate at the time between 11:40 and 12:40 was highest on account of the maximum sunlight intensity (Fig. 11c). This study demonstrated that the output voltage of TE device was able to drive the H_2_ production from water splitting by coating nanomaterials with photoabsorbing and photo-to-heat conversion properties on the hot end of TE devices [156]. Inspired by the dual model effects of surface plasmon resonance (SPR) photothermal conversion and efficient electrocatalytic activity for group VIII metals, our group then proposed Ni nanosheets array grown on the surface of TE device for electrolysis of water. In the integrated device, Ni nanosheets array was served as electrocatalysts and light absorption layer. Replacing the conventional power supply, the output voltage of this integrated system could be immediately applied for electrolysis of water. It was demonstrated that the Ni nanosheets array was utilized as an effective photothermal conversion layer to generate temperature difference (Δ*T*) for TE, and as an efficient electrocatalysts for HER (Fig. 11d, e). The electrolyzer-TE complex devices were constructed for overall water splitting in a two-electrode system, with a H_2_ and O_2_ production rate of 1.818 and 0.912 mmol h^−1^, respectively (Fig. 11f). The integrated TE device provided great advantages for constructing the water splitting system, which were conducive to utilize the solar thermal energy and the waste heat in the prospective applications [157].

### By Integrated Photoelectrochemical-Thermoelectric Device

**Fig. 12:**
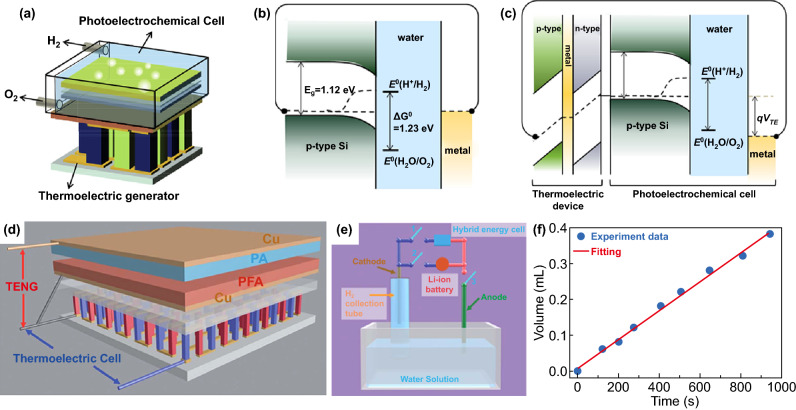
**a** Schematic diagram of a PEC-TE hybrid device. Energy band diagrams of **b** sole PEC and **c** a PEC-TE hybrid circuit under illumination depicting the influence of *qV*_TE_. Reproduced with permission from Ref. [162]. Copyright 2015 Elsevier Inc., **d** schematic diagram of the fabricated TENG-TE hybrid energy cell, **e** schematic diagram of the self-powered system for water splitting to produce H_2_, **f** produced volume of H_2_ at different working times. Reproduced with permission from Ref. [164]. Copyright 2013 Royal Society of Chemistry

The parameters that effect TE conversion efficiency are the Seebeck coefficient (or thermopower), the electrical conductivity and the thermal conductivity [158]. Considering these factors, the energy conversion efficiency of TE device (5–10%) is low, compared to PVs (up to 46%) [159]. Therefore, several researches have demonstrated that by combing TE and PEC reaction, the utilization efficiency for both solar energy harvest and water splitting can reach a high content [160]. The first proposed the combination of PEC and photothermal-electrochemical cycles for H_2_ production by solar energy was by Nikola Getoff in 1984 [161], which was in acid aqueous solution using I_2_ and I_3_^−^ acting as a sensitizer with the existence of ferrous ions. With the introduction of the TE device, the unabsorbed light was collected to provide heat for partly converting into electricity by the TE device. Hence, the efficiency of H_2_ production increased of 30% compared with that of single PEC cycle. However, no research for photothermal-electrochemical water splitting was conducted for a long time since then, until 2015, Lee’s group [126, 162] continuously published two articles to study the integrated PEC-TE device for water splitting by using solar energy and waste heat energy to generate storable and transportable H_2_ fuel. By using the hybrid water splitting device water splitting system, the power generation for H_2_ evolution of 55 mW cm^−2^ was achieved, which was almost 4 times higher than that of a sole PEC cell. According to the total charge transferred, the measured volume of H_2_ was well consistent with the theoretical value of 100% Faraday efficiency (FE), indicating that the generated charge was completely involved in promoting H_2_ evolution. Furthermore, this hybrid operation did not need to use noble metals (e.g., platinum or iridium) because the thermovoltage sole could counteract the kinetic overpotential [126]. Meanwhile, the author explained the enhancement of water splitting in terms of adjusting the Fermi level of the counter electrode with Δ*T* (Fig. 12a–c). As we all known, band edge potentials of semiconductors must straddle the redox potentials of H_2_ and O_2_ for full PEC operation without external bias. However, since the minimum valence band of silicon was not enough positive to oxidize water, silicon was not suitable as a semiconductor material for spontaneous water splitting (Fig. 12b) [162]. Thus, since a TE device was concatenated to a PEC cell, the Fermi level of counter electrode could be adjusted by the applied V_TE_. When the working electrode (p-Si) and counter electrode (Pt) were linked to the positive and negative terminal of the TE device, respectively, electrons injected from the Pt counter electrode flew through the wire to the anode of the TE device. It was worth noting that the Fermi energy level moved downward to a more positive potential until the Fermi energy level of Pt was in alignment with that of the TE device (Fig. 12c). Since the Fermi level of the metal was lower than the oxidation potential of the water, the water would spontaneously oxidize under the action of the *V*_TE_ [162]. Wang et al. proposed a novel PV-TE hybrid device consisted of a serial DSSC, a solar selective absorber (SSA) and a TE generator, providing some inspiration for the development of high-performance PV-TE hybrid devices. The author proposed that the sunlight could be separated into two beams, and UV–visible light was absorbed by a solar cell and infrared light was absorbed by a TE generator to convert into electricity in this hybrid device, which improve the overall conversion efficiency greater than 13%. And thereafter, water splitting driven by TE device integrated PV cell or other electric generation system was widely studied by researchers [163]. Wang’s group manufactured a hybrid energy cell integrated by a TENG, a solar cell and a TE device, which could be utilized for concurrently/separately harvesting mechanical, solar and/or thermal energies. The output power of the hybrid energy cell could be immediately utilized to split water with no external power supply (Fig. 12d–f). The volume of the H_2_ production was linearly related to the splitting time at a generating rate of 4 × 10^−4^ mL s^−1^ (Fig. 12f). As shown in Fig. 12e, there were two ways to water splitting. After the point “1” was concatenated to the point “3,” this hybrid energy cell could be immediately utilized for water splitting, in which the solar cell is in parallel with the rectified TENG. For another way, after the point “1” connecting to the point “2,” the generated energy could be stored in the Li-ion battery and then utilized for electrolysis of water [164].

Intensive research has been conducted to combine PEC cell and TE device for improved solar H_2_ production. However, all of these studies adopt the strategy of connecting PV cells, TE devices and water splitting electrodes together in series. The resulting structure is very complex and not integrated. Therefore, it is a qualitative leap to study how to realize the integration of photodriving components and water splitting components, no matter for effective use of solar energy or for water splitting. The research for integrated device will offer enormous advantages in the aspect of designing the overall water splitting system with integrated structure which are conducive to the practical applications. In addition, the development of an excellent hybrid device which can realize long-term durability of solar water splitting will also become a top priority in the further studies.

## Water Splitting Driven by a Triboelectric Nanogenerator

**Fig. 13:**
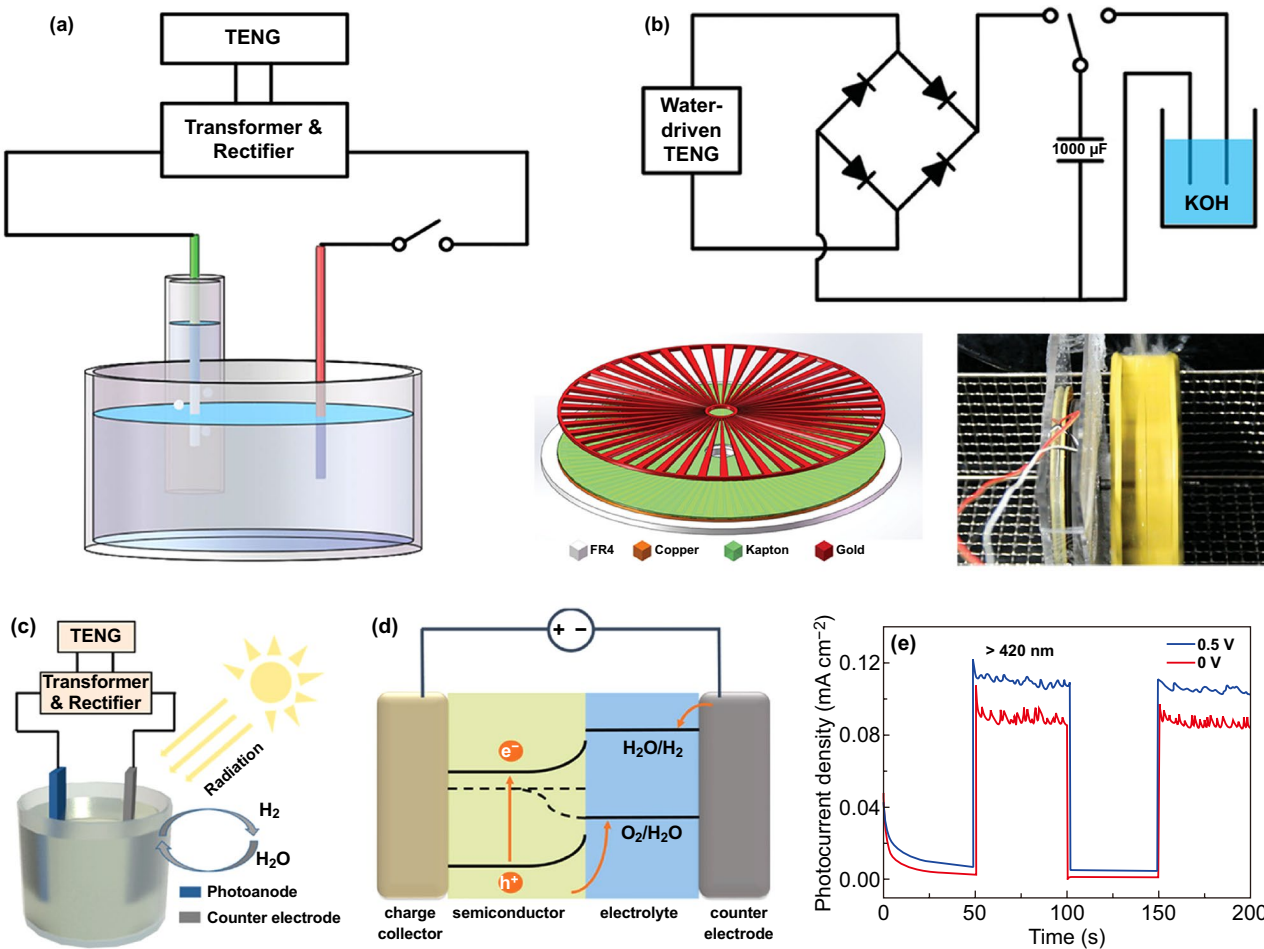
**a** Schematic diagram of the TENG-driven water splitting system, **b** the circuit diagram of the splitting system and the structure of disk TENG. Reproduced with permission from Ref. [172]. Copyright 2014 John Wiley and Sons, **c** schematic diagram of the TENG-PEC hybrid cells, **d** illustration of simplified energy diagram for n-type semiconductor-based solar water splitting, **e** photocurrent of as-prepared Au-decorated TiO_2_ with different bias. Reproduced with permission from Ref. [173]. Copyright 2017 John Wiley and Sons

As depicted in Sects. 4 and 5, water splitting driven by a hybrid energy cell including a PV cell or a TE cell (Fig. 12) paves the way for water splitting driven by other energy devices. Ever since the discovery of TENG by Wang’s group in 2012, TENG had been utilized as an external power supply for water splitting [165, 166]. In terms of energy conversion of TENG, the transfer of contact-induced charges between two triboelectric materials with opposite polarity produced a potential difference during the separation of them [167–171]. Then the produced potential difference would prompt the flow of electrons/ions in the external circuit; hence, it could be utilized as power source [168, 172–178]. In 2014, Tang et al. [172] developed a self-powered hybrid system by combining a water-driven TENG with a water splitting cell (Fig. 13a). The circuit diagram of the splitting system and the structure of disk TENG are shown in Fig. 13b. When the rotated speed of the assembled TENG was 600 rpm, the formation rate of H_2_ in the system reached to 6.25 × 10^−3^ mL min^−1^ in the 30 wt% KOH solution. This research provided a strategy of TENG-driven water splitting for H_2_ generation without external power source. In 2017, the same group prepared a connected TENG-PEC hybrid cell based on a TiO_2_ photoanode, utilizing a flexible TENG to collect environmental dynamic energy, and then charging the Li-ion battery to drive water splitting (Fig. 13c, d) [173]. In the meanwhile, this research proved that the electric field provided by TENG-charged battery played an important role in electrolysis, as well as improved the utilization efficiency of solar energy by boosting the photocurrent (Fig. 13e). Therefore, the TENG-PEC hybrid cell provided an easy and effective method to synergistically transform mechanical and solar energy into chemical energy. Coincidentally, Zhong et al. also developed a self-powered PEC water decomposition system that was combined with a rotatory disk-shaped TENG (RD-TENG), while a titanium modified hematite (Ti–Fe_2_O_3_) was used as the photoanode [168]. It is noted that different rotation speed of TENG had different effects on the output peak current change under illumination and in dark. When at a low rotation speed, the peak current under illumination prominently increased in comparison with that in the dark, while no significant variety at a high rotation speed, indicating the direct electrolysis of water at a high speed.

**Fig. 14:**
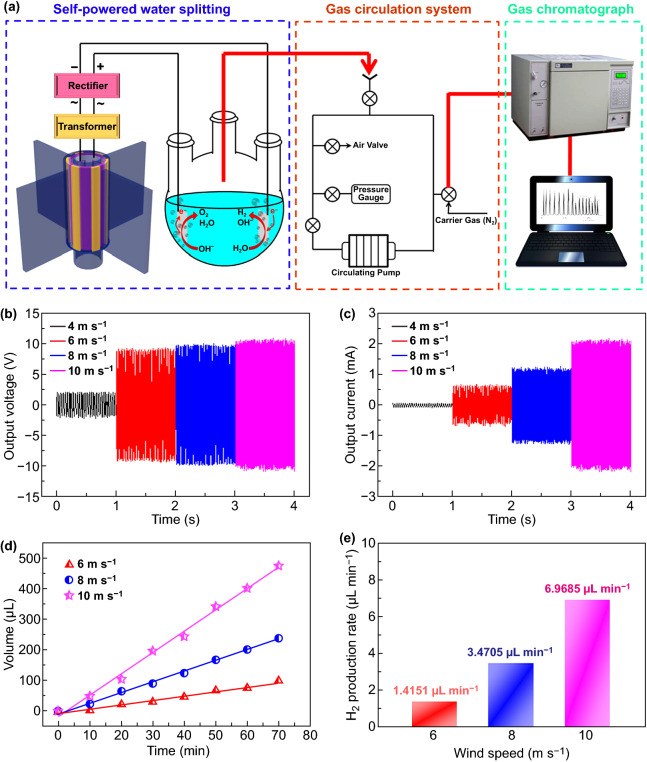
**a** Schematic diagram of CRF-TENG wind energy harvester driven self-powered water splitting system, **b** the output voltage and **c** the output current of the CRF-TENG wind energy harvester with a transformer at different wind speeds, **d** H_2_ production volume as a function of the work times, **e** H_2_ production rate of the CRF-TENG with transformer at different wind speeds. Reproduced with permission from Ref. [12]. Copyright 2018 Elsevier Inc

Besides the water-driven TENG, wind-driven TENG was also widely investigated [179–183]. For example, Fan et al. demonstrated a coaxial rotatory freestanding TENG (CRF-TENG) for collecting wind energy using electrospinning polyvinylidene fluoride (PVDF) nanofiber membrane as triboelectric material (Fig. 14a–c) [12]. And on this basis, a fully self-powered system based on CRF-TENG for water splitting to produce H_2_ was proposed. When the wind speed was 10 m s^−1^, the H_2_ production rate reached 6.9685 μL min^−1^ in 1.0 M KOH solution (Fig. 14d, e).

## Water Splitting Driven by Other Devices

**Fig. 15:**
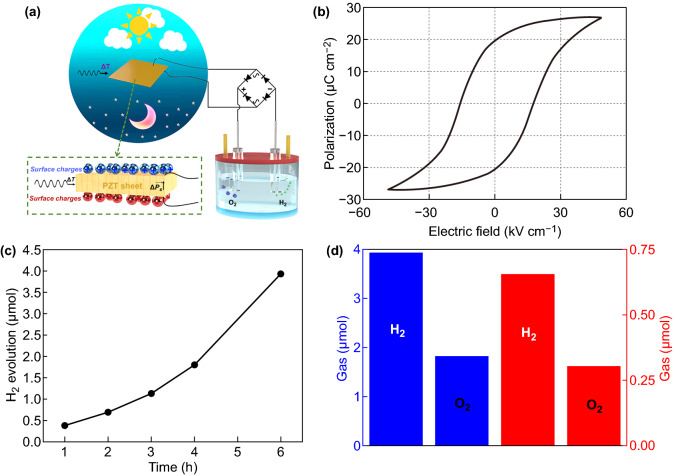
**a** Schematic of pyroelectric as an external source for water splitting, **b** polarization–electric field loop of PZT sheet and schematic of the surface pyroelectric charges, **c** H_2_ evolution from external pyroelectric water splitting with working time from 1 to 6 h, **d** the amount and evolution rate of H_2_ and O_2_ after 6 h detected from the gas chromatography. Reproduced with permission from Ref. [185]. Copyright 2020 Elsevier Inc

Recently, water splitting driven by pyroelectric element attracts much attention for it provides an alternative approach to generate H_2_ from instantaneous low-grade waste heat or natural temperature variations [184–188]. For instance, Xie and Brown et al. proposed to apply pyroelectric effect to produce a large enough electric potential between two electrodes for water splitting into H_2_ and O_2_ gas. The materials utilized in the pyroelectric water splitting system were lead zirconate titanate (PZT-5H) and PVDF thin film [184]. Zhang et al. proposed a pyroelectric water splitting system by utilizing bulk lead PZT as an external charge supply that underwent hot–cold thermal cycles. The schematic diagram of the device was utilized to realize the water splitting with the externally positioned pyroelectric materials (Fig. 15a). As known, the change of ferroelectric polarization with time during thermal cycling was the driving force for the generation of pyroelectric charge during hot–cold fluctuations. Thus, the influences of the electrolyte concentration and heating–cooling frequency on the performance of pyroelectric H_2_ generation were studied (Fig. 15b–d). As demonstrated, the thickness and the area of PZT sheet played an important role in driving water splitting, where the thickness could be used to guarantee an enough potential to initiate water splitting and the area should be maximized to collect the maximal amount of available surface charge [185]. Therefore, future work could concentrate on the formation of pyroelectric nanostructures to enlarge the surface area of the pyroelectric element or exploring the high heat transfer rates of other pyroelectric materials to increase the magnitude and speed of temperature changes [189–192].

**Fig. 16:**
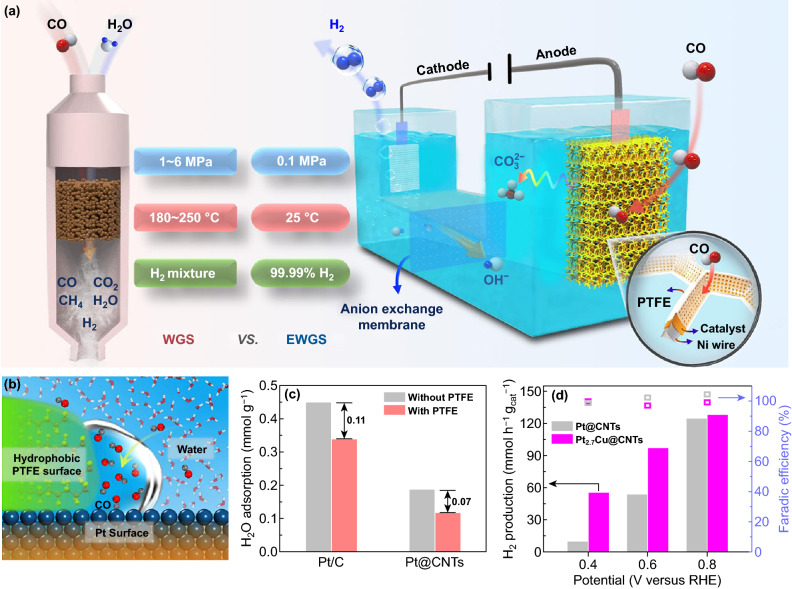
**a** Schematic illustration of the EWGS process in comparison with the traditional WGS process, **b** schematic diagram of solid/liquid/gas interfaces on the PTFE-decorated Pt surface, **c** adsorption of H_2_O at 25 °C on Pt/C and Pt@CNTs with and without PTFE treatment detected by intelligent gravimetric analyzer, **d** the rate of H_2_ production and FE on the cathode at different potentials with the Pt_2.7_Cu@CNTs and Pt@CNTs as the anode catalysts. Reproduced with permission from Ref. [205]. Copyright 2019 Springer Nature

WGS reaction was a main way for industrial H_2_ production [193–199]. For the traditional WGS reaction, high temperatures and high pressures were essential, and H_2_ contamination containing CO_2_, CH_4_ and residual CO was inevitable [200–204]. Herein, Bao et al. reported a novel electrochemical water–gas shift (EWGS) process for directly producing H_2_ with the purity of over 99.99% and the FE of approximately 100% under mild conditions. In contrast to the electrocatalytic water splitting, this WGS reaction afforded a promising alternative way to produce with very low operating voltage, which was realized by the rational design of electrolytic cell and electrocatalysts. In the WGS reaction process of electrolytic cell, CO was oxidized on the anode and H_2_ was produced from H_2_O reduction on the cathode (Fig. 16a). Meanwhile, anion exchange membrane was used to separate the cathode and anode, maintain the balance of electrolyte ion concentration and prevent the cross-contamination of the anodic (CO_2_) and cathodic (H_2_) reaction products in the system. Through optimization of the anode structure by the hydrophobic PTFE layer on catalyst and design of the anode Pt_3_Cu catalyst, the water-free compartments at the interface of PTFE and catalyst to facilitate the diffusion of CO and weaken interaction between CO and anode catalyst surface by Cu were performed (Fig. 16b–d). Finally, directly producing H_2_ with the purity of over 99.99% and the FE of approximately 100% under mild conditions by this novel electrolytic cell was realized [205].

$$\begin{array}{*{20}l} {{\text{Anode}}{:}\;{\text{CO}} + 4{\text{OH}}^{ - } \to {\text{CO}}_{3}^{2 - } + 2{\text{H}}_{2} {\text{O}} + 2e^{ - } } \hfill \\ {{\text{Cathode}}{:}\;2{\text{H}}_{2} {\text{O}} + 2e^{ - } \to {\text{H}}_{2} + 2{\text{OH}}^{ - } } \hfill \\ {{\text{Total}}{:}\;{\text{CO}} + 2{\text{OH}}^{ - } \to {\text{H}}_{2} + {\text{CO}}_{3}^{2 - } } \hfill \\ \end{array}$$

## Outlook and Future Challenges

H_2_ is one of the most sustainable and environmental-friendly energies for replacing fossil fuel energy to mitigate the growing serious energy crisis. In this review, a variety of green energy systems developed for efficient H_2_ production are summarized. The matured two-electrode electrolysis of water system can realize overall water splitting with a high performance at a low cell voltage and long-term stabilities, due to the massive efforts on designing and developing of the bifunctional electrocatalysts with excellent electrocatalytic performance. And many green systems containing photoelectrodes, solar cells, TE devices, TENG devices, pyroelectric devices, and EWGS devices can efficiently utilize renewable energy for water splitting with lower or even no external power source. Therefore, the development of green energy system is significant for utilizing the renewable energy for water splitting.

Although many important developments have been made for green energy systems powered water splitting, this field also faces some challenges. Firstly, most non-noble metal bifunctional catalysts for water splitting show excellent performance only in the alkaline electrolytes, while rare low-cost catalysts for water splitting can work well in the acidic electrolytes. With the introduction of PEM in acidic electrolytes, the utilization of low-cost catalysts for water splitting is attractive. Therefore, the development of highly active non-noble metal catalysts for HER and OER in PEM water electrolyzer is the key thing that needs to be strengthened. Secondly, many developed low-cost catalysts for alkaline water splitting are unable to meet the requirements of high current density and long-term stability in industrial applications. As a consequence, the development of high stability, abundant active sites and large size of electrode for HER and OER is crucial for industrial applications. Thirdly, utilizing the photovoltaic device/TE devices/pyroelectric devices/TENG devices to convert solar energy/thermal energy/wind energy/water energy to electrical energy for delivering water splitting is a promising way to achieve renewable energy driven H_2_ production. However, the photovoltaic device/TE devices/pyroelectric devices/TENG devices in tandem with an electrolyzer will obviously increase the cost for H_2_ production. Therefore, improving the compatibility of devices and the integrity of systems by integrated the photovoltaic device/TE devices/pyroelectric devices/TENG devices with an electrolyzer into a single system will decrease the overall cost for H_2_ production in future practical application.

During the past few years, the different driven systems for water splitting have made great progress and many exciting achievements. With the incessant efforts that are being devoted to this field, water splitting driven by green energy systems will make a significant contribution to large-scale practical applications of clean energy systems in the near future. We hope this review will encourage more efforts into the development of novel green energy system for hydrogen energy production to realize the whole process with low cost, pollution-free and energy sustainability conversion in practical applications.
